# Predicting the 10-year incidence of dyslipidemia based on novel anthropometric indices, using data mining

**DOI:** 10.1186/s12944-024-02006-2

**Published:** 2024-01-31

**Authors:** Alireza Takhttavous, Maryam Saberi-Karimian, Somayeh Ghiasi Hafezi, Habibollah Esmaily, Marzieh Hosseini, Gordon A. Ferns, Elham Amirfakhrian, Mark Ghamsary, Majid Ghayour-Mobarhan, Maryam Alinezhad-Namaghi

**Affiliations:** 1 Student Research Committee, Mashhad University of Medical Sciences, Mashhad, Iran; 2 Department of Nutrition, Faculty of Medicine, Mashhad University of Medical Sciences, Mashhad, Iran; 3 International UNESCO Center for Health-Related Basic Sciences and Human Nutrition, Mashhad University of Medical Sciences, Mashhad, Iran; 4 Endoscopic and Minimally Invasive Surgery Research Center, Mashhad University of Medical Sciences, Mashhad, Iran; 5 Social Determinants of Health Research Center, Mashhad University of Medical Sciences, Mashhad, Iran; 6 Department of Biostatistics, School of Health, Mashhad University of Medical Sciences, Mashhad, Iran; 7 School of Public Health, Department of Epidemiology and Biostatistics, Isfahan University of Medical Sciences, Isfahan, Iran; 8 Brighton & Sussex Medical School, Division of Medical Education, Falmer, Brighton, Sussex BN1 9PH UK; 9 School of Public Health, Department of Epidemiology and Biostatistics, Loma Linda University, Loma Linda, USA; 10 Transplant Research Center, Clinical Research Institute, Mashhad University of Medical Sciences, Mashhad, Iran

**Keywords:** Dyslipidemias, Body surface area, Body mass index, Anthropometry, Data mining, Decision tree

## Abstract

**Background:**

The aim was to establish a 10-year dyslipidemia incidence model, investigating novel anthropometric indices using exploratory regression and data mining.

**Methods:**

This data mining study was conducted on people who were diagnosed with dyslipidemia in phase 2 (*n* = 1097) of the Mashhad Stroke and Heart Atherosclerotic Disorder (MASHAD) study, who were compared with healthy people in this phase (*n* = 679). The association of dyslipidemia with several novel anthropometric indices including Conicity Index (C-Index), Body Roundness Index (BRI), Visceral Adiposity Index (VAI), Lipid Accumulation Product (LAP), Abdominal Volume Index (AVI), Weight-Adjusted-Waist Index (WWI), A Body Shape Index (ABSI), Body Mass Index (BMI), Body Adiposity Index (BAI) and Body Surface Area (BSA) was evaluated. Logistic Regression (LR) and Decision Tree (DT) analysis were utilized to evaluate the association. The accuracy, sensitivity, and specificity of DT were assessed through the performance of a Receiver Operating Characteristic (ROC) curve using R software.

**Results:**

A total of 1776 subjects without dyslipidemia during phase 1 were followed up in phase 2 and enrolled into the current study. The AUC of models A and B were 0.69 and 0.63 among subjects with dyslipidemia, respectively. VAI has been identified as a significant predictor of dyslipidemias (OR: 2.81, (95% CI: 2.07, 3.81)) in all models. Moreover, the DT showed that VAI followed by BMI and LAP were the most critical variables in predicting dyslipidemia incidence.

**Conclusions:**

Based on the results, model A had an acceptable performance for predicting 10 years of dyslipidemia incidence. Furthermore, the VAI, BMI, and LAP were the principal anthropometric factors for predicting dyslipidemia incidence by LR and DT models.

**Supplementary Information:**

The online version contains supplementary material available at 10.1186/s12944-024-02006-2.

## Introduction

Dyslipidemia is impaired lipid metabolism defined as increased levels of total cholesterol (TC), triglycerides (TG), and low-density lipoprotein cholesterol (LDL-C) and decreased levels of high-density lipoprotein cholesterol (HDL-C), that established as an independent predictor of cardiovascular and cerebrovascular occurrences [1, 2]. Cardiovascular disease (CVD) is responsible for substantial declines in both the quality of life and lifespan, while simultaneously imposing a considerable economic strain on healthcare systems worldwide [3]. The prevalence of dyslipidemia depends on geography; however, it has been estimated that more than 50% of adults (non-Hispanic white, non-Hispanic black, Mexican American, and urban and rural India) have dyslipidemia [4–6]. Also, the prevalence of dyslipidemia is noticeable in Iran, according to a meta-analysis that was published in 2014 with 29 articles and one unpublished data, the approximate prevalence of dyslipidemia in Iranian women and men, rural and urban populations for increased plasma concentration of triglyceride, cholesterol, LDL-C respectively were 46%, 41.6%, 35.5%; and for decreased HDL-C was 43.9% [7].

Dyslipidaemia is highly related to obesity [8]; anthropometric parameters have been employed for assessing obesity in both epidemiological and clinical investigations [9]. Significant differences in anthropometric parameters have been observed between individuals with dyslipidemia and those without, indicating a notable difference between the two groups, and a direct relationship between dyslipidemia and Body Mass Index (BMI) has been reported [10, 11], however, there are some controversies [12]. Novel anthropometric indices have been introduced for a better description of body shaping during the last decades [13–15]. It has been claimed that some of them are associated with all causes of mortality risk [16], cardio-metabolic risk [14], impaired glucose tolerance [17], and type 2 diabetes mellitus (T2DM) [18] dyslipidemia [19], but there are some controversies [20].

Rodolfo Valdez et al., introduced the Conicity Index (C-Index) to evaluate obesity [13]. Thomas et al. suggested the Body Roundness Index (BRI) as a predictor of visceral adiposity tissue that can be used to evaluation of health status [15]. Visceral Adiposity Index (VAI) can be calculated from waist circumference [21] and BMI, along with assessing TG and HDL-C. So it is a convenient index for evaluating visceral fat dysfunction. As such, VAI could be beneficial in clinical practice, population studies, and estimating the cardio-metabolic risk linked to visceral obesity [14]. Lipid Accumulation Product [22] proved to be more efficacious than BMI for discerning United States adults at risk for CVD and it was defined by two measurements waist circumference (WC) and TG [23]. Abdominal Volume Index (AVI) has been demonstrated as a reliable and convenient anthropometric tool for estimating overall abdominal volume and has a strong correlation with Impaired Glucose Tolerance (IGT) and DM [18]. Also, AVI exhibited significant potential as a diagnostic tool for metabolic syndrome [24]. The association between AVI and atherogenic dyslipidemia has been reported [19].

A Body Shape Index (ABSI) has been developed as a new indicator [25]. It was a strong predictor for all causes of mortality [16]. One study showed that the reliability of this index and experimental measurement of lipid profile can be equal [26]. Body Adiposity Index (BAI) is a formula that estimates the adiposity of people without using weight [27]. Also, Weight-Adjusted-Waist Index (WWI) is a promising alternative marker for obesity and is associated with adverse health consequences [28]. Values pertaining to the Body Surface Area (BSA) are frequently employed within the medical domain, primarily for the purpose of computing the dosages of chemotherapeutic agents and determining the index of cardiac output [29].

To the best of our knowledge, there is no other predictive model for dyslipidemia incidence prediction, based on novel anthropometric indicators using artificial intelligence. According to the high prevalence of dyslipidemia and its relation to CVD, this study aimed to establish a 10-year predicting model for dyslipidemia based on novel anthropometric indices, using exploratory regression models and data mining.

## Methods

### Study population

**Fig. 1:**
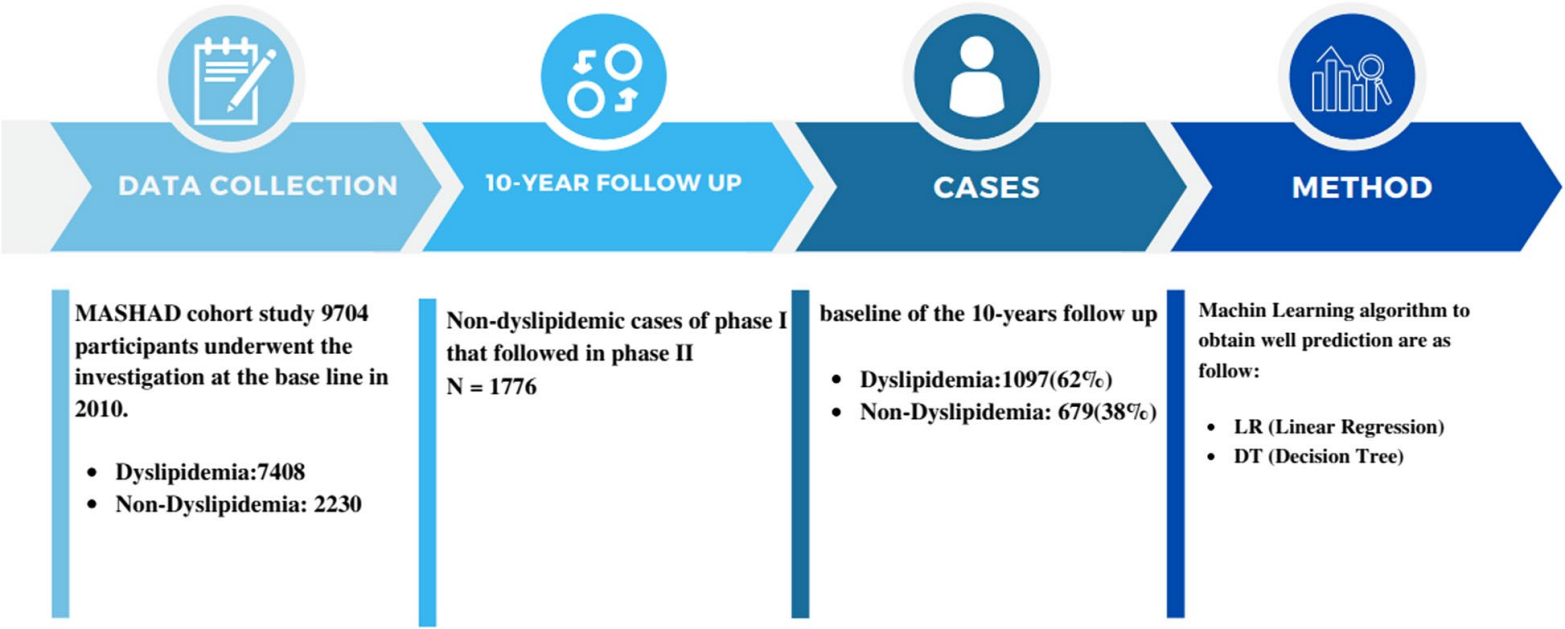
The study flowchart

This cohort study was conducted on all non-dyslipidemia participants from the Mashhad stroke and heart atherosclerotic disorder (MASHAD) cohort study; a 10-year cohort from northeastern Iran, in which all participants signed the written consent forms [30]. Among 9704 subjects of first phase of the MASHAD study, 2230 people did not have dyslipidemia. Of those, 1776 were evaluated in Phase 2 follow-up in 2019 (Fig. 1). The study protocol was approved by the Ethical Committee of Mashhad University of Medical Sciences (ID = IR.MUMS.MEDICAL.REC.1402.262).

### Baseline examination

Dyslipidemia was defined as a serum TC <200 mg/dl (5.18 mmol/l), LDL-C 130 mg/dl (3.36 mmol/l), or TG 150 mg/dl (1.69 mmol/l), or HDL-C <40 mg/dl (1.03 mmol/l) in men and <50 mg/dl (1.30 mmol/l) in women [31]. If serum TG concentrations were lower than 400 mg/dl; LDL-C was computed from the serum TG, TC, and HDL-C concentrations expressed in mg/dl using the Friedewald formula [32].

A registered nurse measured anthropometric measurements such as weight, height, Hip Circumference (HC), Mid-arm Circumference (MAC), and WC. Waist-to-hip Ratio (WHR), BMI, BAI, C-Index, BRI, WWI, AVI, Lipid Accumulation Product (LAP), VAI, and BSA were computed utilizing the formulas presented in the Table of the Supplementary Appendix 1. Within measurements of height and weight wanted participants to wear light clothes and no shoes. According to the World Health Organization recommendations, a BMI between 25 and 29.99 kg/m^2^ was categorized as overweight and a BMI≥30 kg/m^2^ was classified as obese.

A current smoker was identified as one who smokes cigarettes daily, whereas an ex-smoker was defined as an individual who previously smoked daily, but no longer does so, and non-smoker subjects were acquired. Psychometric tests were conducted with Beck’s anxiety inventory to calculate an anxiety score, which was interpreted as follows: 0-7, minimal level of anxiety; 8-15, mild anxiety; 16-25, moderate anxiety; and 26-63, severe anxiety. The Beck’s Depression Inventory-II (BDI-II) was also employed to evaluate depression scores, with the following cut-offs: 0-13, minimal depression; 14-19, mild depression; 20-28, moderate depression; and 29-63, severe depression.

### Statistical analysis

All data were analyzed using the R Statistical Software (v4.1.2; R Core Team 2021), The IBM SPSS Statistics (Version 27), and MedCalc statistical software version 13. All continuous data are expressed as mean ± SD and frequency (%) for categorical. All *P*-value < 0.05 was regarded as statistically significant. We used a t-test for continuous variables, and the Mann–Whitney test for non-normal data was employed to compare the mean or median of subjects with and without Dyslipidemia. The chi-square test was implemented to investigate the association between the categorical variables and the binary outcome, dyslipidemias. Data was split into train and test datasets using the holdout method as 75% and 25 % respectively.

Logistic Regression (LR) was performed with dyslipidemia incidents as the response variable and anthropometric factors. Some of these variables have collinearity, which in LR method were eliminated. To assess the multicollinearity between independent variables the variance inflation factor (VIF) as well as the computation of correlation coefficient was used. Generally, a correlation higher than 0.95 was considered a highly correlated variable that helps to recognize the possibility for multicollinearity. The logistic regression was used to compute the odds ratios (OR) with their 95% confidence interval. To compare the models and study the model’s goodness of fit test, the deviance as a likelihood ratio statistic was applied.

### Decision tree model

The data put into a data mining approach and the Decision Tree (DT) was drawn to form a predictive model of anthropometric measurements. A decision tree is a non-parametric method named regarding the nature of the target variable. The aim of a decision tree is to form a predictive model in terms of predictor variables. This paper uses the CHIAD technique in DT. CHAID can be used for prediction as well as classification, and for detection of interaction between variables. There is a different algorithm written to assemble a decision tree, which can be utilized by the problem a few of the commonly used algorithms are (CART, ID3, C4.5, and CHAID). To find the most dominant feature, chi-square tests will use that is also called CHAID.

Data mining techniques were used to investigate the relationship between anthropometric predictors and binary response variables (dyslipidemia, and non-dyslipidemia). So, the main objective of this study was to anticipate dyslipidemias using the DT model and to determine their associated factors, especially anthropometric markers. For this purpose, the dataset was randomly split into two parts: training data, and test data (25%-75%). The training dataset was utilized to develop the DT model, which was then validated using test data (25%) that hadn't been used during training. DT algorithms use splitting criteria to break a node into branches to reduce the impurity of a node. This criterion provides a rate for every predictor variable.

## Results

### Characteristics of the study population

From the total study population of 9704 participants in phase 1, about 1776 non-dyslipidemia individuals whom were followed in phase 2 (10-year follow-up) enrolled to the study. As shown in Fig. 1, a total of 1097 subjects were dyslipidemic in the second phase of the study.

Table 1 shows that 1776 participants consisted of 679 with non-dyslipidemia and 1097 individuals suffered from dyslipidemia during phase 2 of the study. There were 876 (49.3%) females of whom 586 (53.4%) had dyslipidemia and 900 males (50.7%) of those 511(46.6%) had dyslipidemia. The serum glucose level was significantly higher in subjects with dyslipidemia than in non-dyslipidemias (*P* = 0.002). It is noticeable that close to 60% were overweight (37.3%) or obese (19.2%) with a significantly higher rate of dyslipidemia in overweight (39.1% vs 34.3%) and obese (22% vs 14.6%) participants. Apparently, smoking had no significant association with dyslipidemia (*P* = 0.949). The variables C-Index, BRI, VAI, LAP, and AVI all showed significantly higher in subjects with dyslipidemia compared to individuals without dyslipidemia (*P* < 0.001), and the rest of continuous variables were non-significant differences between the two groups.

**Table 1:** Description of characteristics of study participants

Variable	Total (*n* = 1776)	Non Dyslipidemia (*n* = 679)	Dyslipidemia (*n* = 1097)	*P*-value
C-Index	1.30 ± 0.12^a^	1.29 ± 0.11	1.31 ± 0.12	< 0.001
BRI	4.91 ± 1.88	4.53 ± 1.71	5.14 ± 1.94	< 0.001
VAI	1.21 ± 0.47	1.05 ± 0.42	1.31 ± 0.47	< 0.001
LAP	31.22 ± 17.91	25.87 ± 15.51	34.58 ± 18.49	< 0.001
AVI	17.27 ± 4.52	16.53 ± 4.26	17.73 ± 4.62	< 0.001
PAL	393.90 ± 74.25	394.40 ± 76.37	393.59 ± 72.94	0.825
WWI	11.16 ± 1.08	11.02 ± 0.99	11.24 ± 1.13	< 0.001
ABSI	0.08 ± 0.01	0.08 ± 0.01	0.08 ± 0.01	0.352
Percent of energy intake from protein	15.45 ± 4.15	15.25 ± 4.09	15.58 ± 4.19	0.175
Percent of energy intake from carbohydrate	52.94 ± 11.26	53.16 ± 11.56	52.80 ± 11.06	0.592
Percent of daily energy intake from fat	34.86 ± 10.10	34.81 ± 10.44	34.90 ± 9.88	0.886
Age	47.36 ± 8.13	47.03 ± 8.34	47.56 ± 7.99	0.181
Anxiety Score	9.57 ± 9.43	9.47 ± 9.22	9.64 ± 9.56	0.715
Depression Score	11.11 ± 8.96	11.08 ± 8.97	11.12 ± 8.96	0.930
BMI	28.05 ± 7.88	29.95 ± 12.72	27.21 ± 4.30	0.371
Glucose	81 (74,90)^b^	81 (73,88)	82 (74, 91)	0.002
BMI				< 0.001
Underweight (< 18)	38 (2.14%)^c^	26 (3.83%)	12 (1.09%)	
Normal (18 – 25)	709 (39.92%)	308 (45.36%)	401 (36.55%)	
Overweight (25 – 30)	662 (37.27%)	233 (34.32%)	429 (39.11%)	
Obese (> 30)	341 (19.20%)	99 (14.58%)	242 (22.03%6)	
Missing	26 (1.46%)	13 (1.91%)	13 (1.19%)	
Smoking status				0.949
Non smoker	1269 (71.45%)	484 (71.25%)	785 (71.56%)	
Ex-smoker	173 (9.74%)	65 (9.57%)	108 (9.85%)	
Current smoker	334 (18.81%)	130 (19.15%)	204 (18.60%)	
Sex				< 0.001
Male	900 (50.68%)	389 (57.29%)	511 (46.58%)	
Female	876 (49.32%)	290 (42.71%)	586 (53.42%)	

*Abbreviations*: *C-Index* conicity index, *BRI* body roundness index, *VAI* Visceral Adiposity Index, *LAP* Lipid Accumulation Product, *AVI* Abdominal volume index, *WWI* weight-adjusted-waist index, *BMI* body mass index, *BAI* Body Adiposity Index, *ABSI* Body Shape Index, *PAL* physical activity level

a. Mean ± sd for continuous and normal variables and *P*-value of two sample t test

b. Median (Q1,Q3) for continuous and abnormal variables and *P*-value of Mann Whitney U test

c. Count (percentage) for categorical variables and *P*-value of chi square test

### The association between anthropometric measurements and dyslipidemia using Logistic Regression (LR) model

**Fig. 2:**
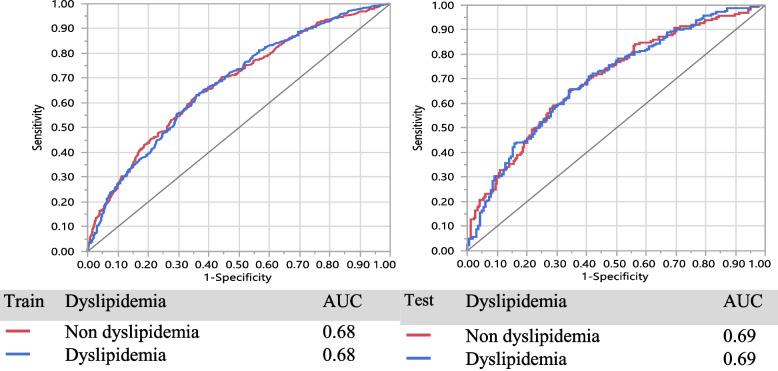
LR method for model A, in Table 2

**Fig. 3:**
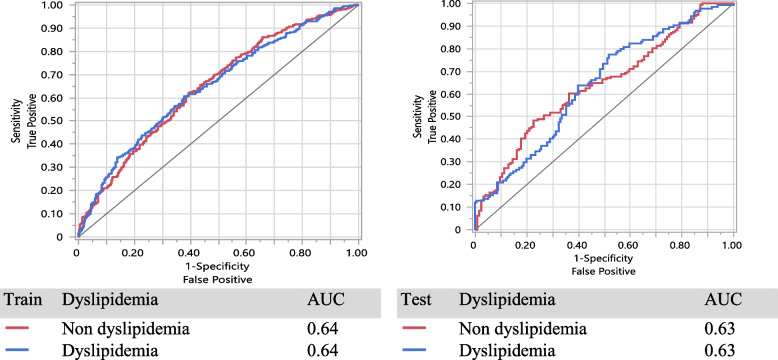
LR method for model B, in Table 2

In Table 2, the result of LR is presented based on two models. Model A, including the variables VAI, BRI, and BMI, and Model B included all variables in Model A adjusted for physical activity level (PAL), age, sex, anxiety score, depression score, smoking status, percent of daily energy intake from fat, percent of energy intake from carbohydrate and percent of energy intake from protein. The LR applied to each anthropometrics variable with inclusion in the model based on a 20% level of significance (see Table 2 crude OR (95% CI)), the results lead to including the variables VAI, BRI, and BMI and they form model A. Furthermore, model B contains all variables in Model A plus the confounder variables explained above. The variable VAI is positively associated with dyslipidemias and significant in all 3 models (*P* < 0.001), but BRI and BMI are only significant in the crude model (*P* < 0.001). Clearly, the variable VAI is the most remarkable predictor of dyslipidemia. In model A, the odds of individuals with dyslipidemia for each unit increasing in VAI is 3.4 times of those without dyslipidemias (OR: 3.40, (95% CI: 2.63, 4.38)) adjusting for BRI and BMI. Similarly in model B, VAI is a significant predictor of dyslipidemias (OR: 2.81, (95% CI: 2.07, 3.81)) adjusting for all other variables. Moreover, it was found that model B has a significantly better fit than model A in terms of deviance (2139.2 vs 1460.6, *P* =  < 0.001). The ROC curve in train and test in model A and model B are shown in Fig. 2 and Fig. 3 respectively. Also, the sensitivity of model A (87% for the train vs 87% for the test) is better than model B (86% for the train vs 81% for the test).

**Table 2:** Association of anthropometric indices with incident dyslipidemia for one standard deviation increase

Variable		Crude OR(95%CI)	*P*-value	Model A OR (95% CI)	*P*-value	VIF Model A	Model B OR (95% CI)	*P*-value	VIF Model B
VAI		3.77 (2.97, 4.80)	< 0.001	3.40 (2.63, 4.38)	0.000	1.11	2.81 (2.07, 3.81)	< 0.001	1.13
BRI		1.21 (1.14, 1.28)	< 0.001	1.05 (0.96, 1.13)	0.287	2.01	1.01 (0.91, 1.13)	0.843	2.55
BMI						1.90			2.44
Underweight (< 18)		0.35 (0.18, 0.71)	0.004	0.40 (0.19, 0.84)	0.015		0.43 (0.18, 1.03)	0.260	
Normal (18 – 25)		Ref	-	Ref	-		Ref	-	
Overweight (25 – 30) Obese (> 30)		1.41 (1.14, 1.76) 1.88 (1.42, 2.48)	0.002 < 0.001	1.09 (0.85, 1.41) 1.17 (0.80, 1.73)	0.496 0.417		1.07 (0.77, 1.49) 0.98 (0.59, 1.63)	0.696 0.941	
Sensitivity	**Train**	-		87%			86%		
	**Test**	-		87%			81%		
Deviance = -2LLR		-		2139.15			1460.57		
*P* value of deviance		-		0.000					

The model A contains VAI, BRI, BMI

The model B contains model all variables in Model A, adjusted for PAL, Age, Sex, Anxiety Score, Depression Score, Smoking status, percent of daily energy intake from fat, percent of daily energy intake from carbohydrate and percent of daily energy intake from protein

*Abbreviations*: *BRI* Body roundness index, *VAI* Visceral Adiposity Index, *BMI* body mass index, *PAL* physical activity level

Supplementary Appendix 2 has summarized the association between anthropometric measurements and dyslipidemia using LR Model.

### The Association between Anthropometric measurements and dyslipidemia using Decision Tree (DT) Model

**Fig. 4:**
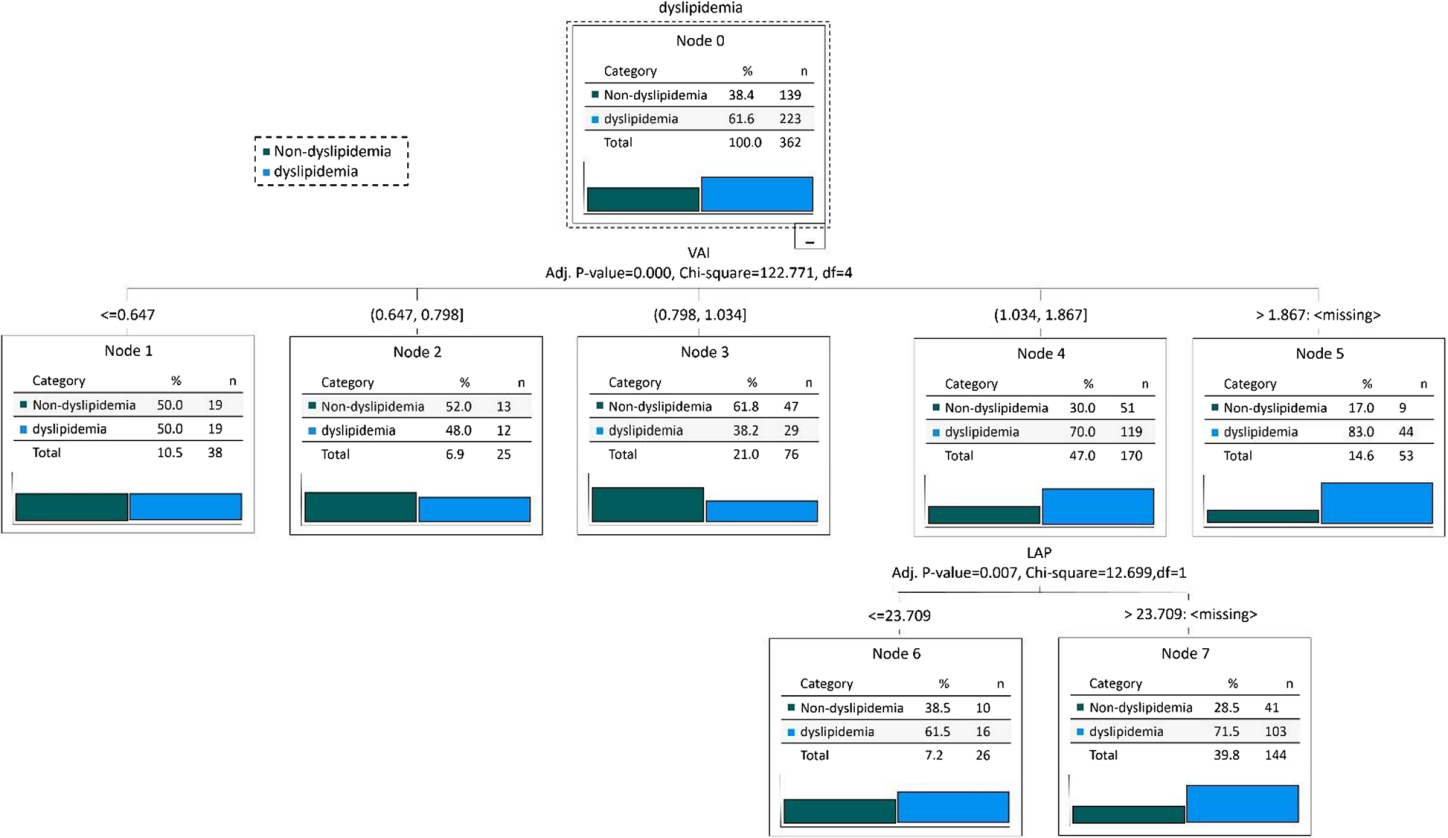
DT for Dyslipidemia without confounding factors. Abbreviations: VAI (Visceral Adiposity Index), BMI (body mass index), and LAP (Lipid Accumulation Product)

**Fig. 5:**
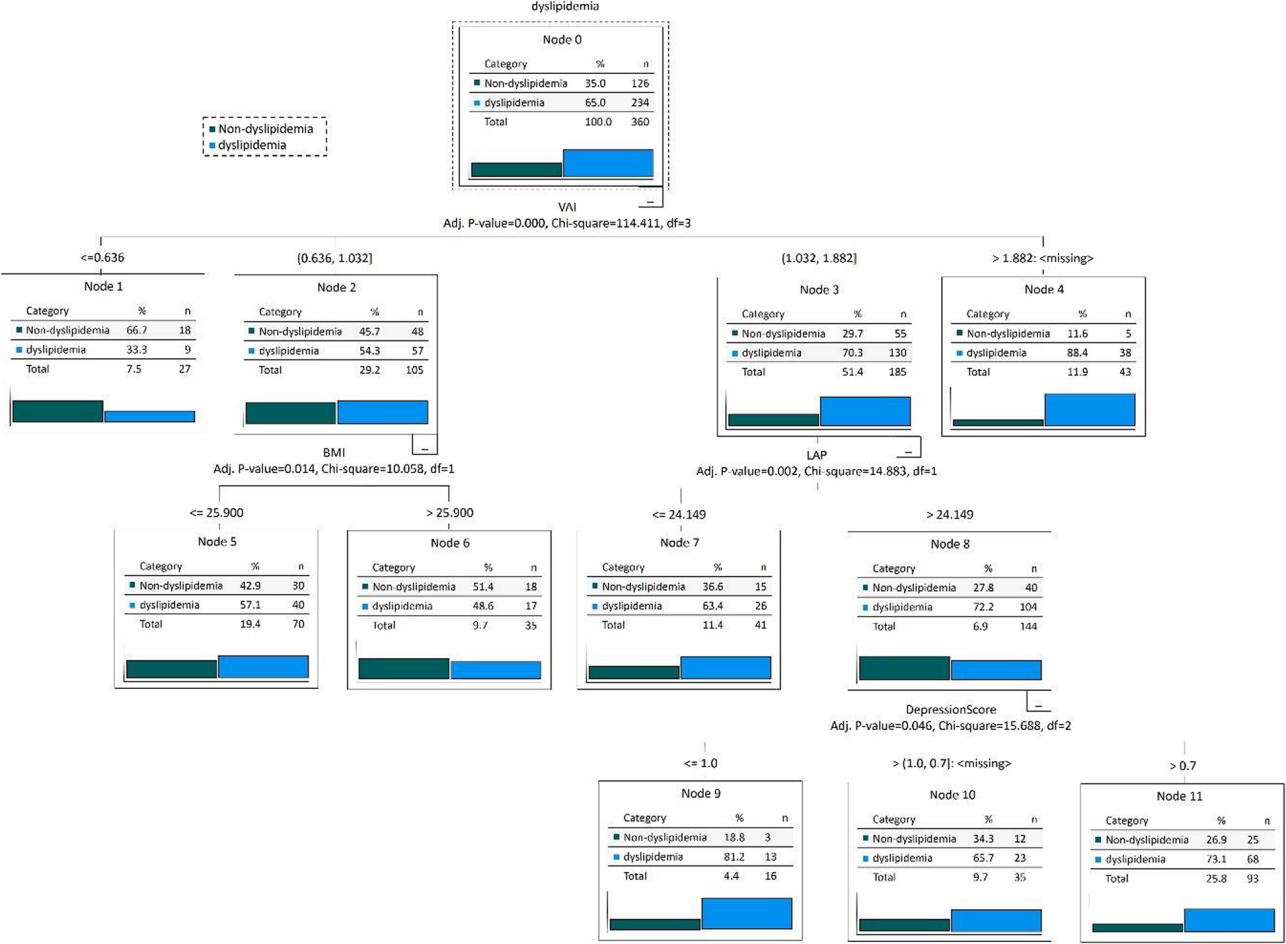
DT for Dyslipidemia with confounding factors. Abbreviations: VAI (Visceral Adiposity Index), BMI (body mass index), and LAP (Lipid Accumulation Product)

Figure 4 and Fig. 5 shows the outcomes of the DT testing for anthropometry factors and anthropometry factors with confounding factors. In the first case, the DT algorithm determined the various dyslipidemia risk factors and categorized them into 2 layers. According to the DT model, the first variable [33] has the highest significance for classifying data, while the subsequent variables have lower significance. Figure 4 illustrates that VAI, followed by LAP, has the greatest impact on the dyslipidemia presence risk. Participants with VAI $\le$ 0.64 had lower dyslipidemia, according to the DT model than those with higher VAI and LAP levels (0.5 vs. 0.5 incident rate). Higher VAI and LAP were more commonly associated with Dyslipidemia. Table 3 illustrates the specific dyslipidemia rules developed by the DT model. Therefore VAI and LAP were thus determined to be the most crucial variables in the DT model and to predict the dyslipidemia incidence.

**Table 3:** DT Rules for Dyslipidemia

Rules	Dyslipidemia (%)	Non- Dyslipidemia (%)
DT Rules for Dyslipidemia without confounder		
R1: VAI < = 0.64	50.00	50.00
R2: VAI > 0.64 & VAI < = 0.79	48.00	52.00
R3: VAI > 0.79 & VAI < = 1.03	38.20	61.80
R4: VAI > 1.03 & VAI < = 1.86 & LAP < = 23.70	61.50	38.50
R5: VAI > 1.03 & VAI < = 1.86 & LAP > 23.70	71.50	28.50
R6: VAI > 1.86	83.00	17.00
DT Rules for Dyslipidemia with confounder		
R1: VAI < = 0.63	33.30	66.70
R2: VAI > 0.63 & VAI < = 1.03 & BMI < = 25.89	57.10	42.90
R3: VAI > 0.63 & VAI < = 1.03 & BMI > 25.89	48.60	51.40
R4: VAI > 1.03 & VAI < = 1.88 & LAP < = 24.14	63.40	36.60
R5: VAI > 1.03 & VAI < = 1.88 & LAP > 24.14 & Depression Score < = 1	81.20	18.80
R6: VAI > 1.03 & VAI < = 1.88 & LAP > 24.14 & Depression Score > 1 & Depression Score < = 7	65.70	34.30
R7: VAI > 1.03 & VAI < = 1.88 & LAP > 24.14 & Depression Score > 7	73.10	26.90
R1: VAI < = 0.63	33.30	66.70

*Abbreviations*: *LAP* Lipid Accumulation Product, *VAI* Visceral Adiposity Index, *BMI* body mass index, *PAL* physical activity level

In the second case (with disturbances) DT algorithm determined the various dyslipidemia risk factors with disturbances and categorized them into 3 layers. According to the DT model, the first variable [33] has the highest significance for classifying data, while the subsequent variables have lower significance. Figure 5 illustrates that VAI, followed by BMI, LAP, and depression score has the greatest impact on the dyslipidemia presence risk. Participants with VAI $\le$ 0.63 had lower dyslipidemia, according to the DT model than those with higher BMI, LAP, and depression score levels (0.333 vs. 0.667 incident rate). Higher BMI and LAP were more commonly associated with dyslipidemia.

Variables with the best rate were selected to be included in the model. Table 4 illustrates the confusion matrix of the decision trees were used to evaluate the accuracy, precision, and specificity.

**Table 4:** Performance indices of the DT model for Dyslipidemia

A: Performance indices of the DT adjusted model for variables without confounding factors					
(a) Training (*n* = 1379)			(b) Testing (*n* = 345)		
**Actual**	**Predicted Count**		**Actual**	**Predicted Count**	
	Non- Dyslipidemia	Dyslipidemia		Non-Dyslipidemia	Dyslipidemia
Non-Dyslipidemia	171	367	Non-Dyslipidemia	32	107
Dyslipidemia	106	767	Dyslipidemia	31	192
Specificity = 87%	AUC = 65%		Specificity = 59%		AUC = 67%
Accuracy = 66%	Precision = 67%		Accuracy = 48%		Precision = 64%
B: Performance indices of the DT adjusted model for variables with confounding factors					
(a) Training (*n* = 1379)			(b) Testing (*n* = 345)		
**Actual**	**Predicted Count**		**Actual**	**Predicted Count**	
	Non-Dyslipidemia	Dyslipidemia		Non-Dyslipidemia	Dyslipidemia
Non-Dyslipidemia	231	322	Non-Dyslipidemia	48	78
Dyslipidemia	151	712	Dyslipidemia	49	185
Specificity = 82%	AUC = 65%		Specificity = 70%		AUC = 67%
Accuracy = 66%	Precision = 68%		Accuracy = 64%		Precision = 79%

## Discussion

Generally, this study has shown that VAI is the most important predictor for the incidence of dyslipidemia in all models. Moreover, BMI and LAP were identified as the most critical variables after VAI in the decision tree model to predict dyslipidemia incidence.

A 10-year predicting model for the incidence of dyslipidemia was established in the current study. To the best of our knowledge, there is no similar study that has a predictive model design, using artificial intelligence and includes novel anthropometric indicators. The results have shown that VAI is a strong predictor for 10-year anticipation of dyslipidemia. Although, BMI and LAP were identified as the most important variables in the prediction of dyslipidemia incidence.

The results of previous studies are in line with current study that introduced VAI as the most powerful prognosticator of atherogenic dyslipidemia [19]. Also, it has been found that LAP was a beneficial factor in predicting the incidence of dyslipidemia. Recently, Zhou, Y et al., showed that LAP was more associated with dyslipidemia than BMI among university staff in a cross-sectional study [34]. It has been stated that the BRI's potential role in predicting dyslipidemia is comparable with BMI but not stronger [35]. It has been shown that BRI can be a good indicator for dyslipidemia prediction in women but not in men [36]. Maybe this difference relates to the diversity of visceral adiposity between genders.

It has been shown that VAI serves as a significant tool for determining insulin sensitivity, and its elevation exhibits a robust correlation with the risk of developing cardio-metabolic disorders [14]. Insulin resistance plays a crucial role in the metabolism of very low-density lipoprotein (VLDL), including its effect of elevating the synthesis of hepatic VLDL triglycerides (VLDL-TGs) [37, 38]. The enhanced synthesis of VLDL-TGs is associated with the escalated production of hepatic Apolipoprotein B-100 (apo B-100) [37–39]. This leads to hypertriglyceridemia, increases in particle number exhibited by VLDL apo B-100 and decreased HDL-C concentrations [39]. Insulin resistance is also associated with an increase in hepatic triglyceride lipase (HTGL), which may result in the accelerated clearance of HDL-C and a reduction in HDL-C [40].

The reason for the difference between previous findings and current study results can be due to the difference in the included indicators of the studies and the statistical methods used.

## Study strengths and limitations

This was a 10-year follow-up cohort study with a large population, using novel anthropometric indices for the prediction of dyslipidemia incidence. Using analytical techniques, including the machine learning algorithms such as the decision tree method and Logistic Regression Model was another robustness of the study. To the best of our knowledge, the design of previous studies was cross-sectional. There is no longitudinal study among subjects without dyslipidemia with a 10-year follow-up.

To explicate these findings the following limitations ought to be acknowledged: Possibly, some individuals with dyslipidemia were geriatric (aged ≥ 65 years); however, current research only comprised participants aged 35 to 65 years old. Also, some factors such as epigenetics, can be included in future studies.

## Conclusion

The present study established a 10-year predicting model for dyslipidemia incidence, for the first time. Generally, current study has shown that in all models VAI is the most important predictor for dyslipidemia incidence. This finding emphasis on the importance of visceral obesity to dyslipidemia incidence and suggest to target the visceral obesity improvement for decreasing the incidence of dyslipidemia.

### Supplementary Information

**Additional file 1: Supplementary Appendix 1.** Computed formula utilizing for calculation of novel anthropometric indices.

**Additional file 2:** **Supplementary Appendix 2.** The association between anthropometric measurements and dyslipidemia using Logistic Regression (LR) Model. **Table 1.Supp.** Best Cut-off values of anthropometric indices maximizing sensitivity plus specificity using receiver operating characteristic analysis. **Table 2.Supp.** Association of the binary anthropometric indices with incident dyslipidemia based on optimal cutoff point. **Figure 1.Supp.** Model A based on optimal cutoff point, in Table 4. **Figure 2.Supp.** Model B based on optimal cutoff point, in Table 4.

## Data Availability

The data will be available upon a reasonable request from the corresponding author.
