# Supplementary material for: Predicting the 10-year incidence of dyslipidemia based on novel anthropometric indices, using data mining
Source: Lipids Health Dis. 2024 Jan 31;23:33. doi: 10.1186/s12944-024-02006-2 (PMC10829243; doi:10.1186/s12944-024-02006-2)
Supplement: Supplementary file 1 — Additional file 1: Supplementary Appendix 1. Computed formula utilizing for calculation of novel anthropometric indices. [file 12944_2024_2006_MOESM1_ESM.docx]

**Supplementary Appendix 1**: Computed formula utilizing for calculation of novel anthropometric indices

| Conicity Index | $\frac{WC(m)}{0.109\times\sqrt{\frac{weight(Kg)}{height(m)}}}$(1) |
| --- | --- |
| BRI | $364.2-365.5\times\sqrt{1-\frac{{(\frac{(WC(m)}{2\pi})}^{2}}{{(0.5\times height\left( m \right))}^{2}}}$(2) |
| VAI | Men:  $\frac{WC\left( cm \right)}{[39.68+(1.88\times BMI\left( \frac{Kg}{m^{2}} \right)]}\times(\frac{TG\left( \frac{mmol}{L} \right)}{1.03}\times(\frac{1.31}{HDL(\frac{mmol}{L})}$(  Women:  $\frac{WC\left( cm \right)}{[39.58+(1.89\times BMI\left( \frac{Kg}{m^{2}} \right)]}\times(\frac{TG\left( \frac{mmol}{L} \right)}{0.81}\times(\frac{1.52}{HDL(\frac{mmol}{L})}$((3) |
| LAP | men = $(WC\left( cm \right)-65)\times(TG(\frac{mmol}{L})$)  women = $(WC\left( cm \right)-58)\times(TG(\frac{mmol}{L}))$(4) |
| AVI | $\frac{[2\times{WC}^{2}\left( cm \right)+0.7\left( WHR \right)^{2}]}{1000}$(5) |
| WWI | $\frac{WC(cm)}{\sqrt{weight(Kg)}}$(6) |
| BAI | $\frac{\mathrm{HC}}{\mathrm{height}^{1.5}}-18$ (7) |
| BSA | $\sqrt{\frac{height(cm)\times weight(kg)}{3600}}$ (8) |
| BMI | $\frac{Weight(kg)}{{height}^{2}(m^{2})}$ |
| WHR | $\frac{WC(m)}{HC(m)}$ |
| Abbreviations: BRI( Body Roundness Index),WC (waist circumference)VAI( Visceral Adiposity Index) LAP (Lipid Accumulation Product),AVI (Abdominal volume index),WWI(weight-adjusted-waist index),BAI (Body Adiposity Index),HC (Hip Circumference ),BSA(body surface area), BMI(Body Mass Index) and WHR( Waist-to-hip Ratio) | |

**References**

1. Valdez R. A simple model-based index of abdominal adiposity. Journal of clinical epidemiology. 1991;44(9):955-6.

2. Thomas DM, Bredlau C, Bosy‐Westphal A, Mueller M, Shen W, Gallagher D, et al. Relationships between body roundness with body fat and visceral adipose tissue emerging from a new geometrical model. Obesity. 2013;21(11):2264-71.

3. Amato MC, Giordano C, Galia M, Criscimanna A, Vitabile S, Midiri M, et al. Visceral Adiposity Index: a reliable indicator of visceral fat function associated with cardiometabolic risk. Diabetes care. 2010;33(4):920-2.

4. Kahn HS. The" lipid accumulation product" performs better than the body mass index for recognizing cardiovascular risk: a population-based comparison. BMC cardiovascular disorders. 2005;5:1-10.

5. Guerrero-Romero F, Rodrı́guez-Morán M. Abdominal volume index. An anthropometry-based index for estimation of obesity is strongly related to impaired glucose tolerance and type 2 diabetes mellitus. Archives of medical research. 2003;34(5):428-32.

6. Park Y, Kim NH, Kwon TY, Kim SG. A novel adiposity index as an integrated predictor of cardiometabolic disease morbidity and mortality. Scientific reports. 2018;8(1):16753.

7. Bergman RN, Stefanovski D, Buchanan TA, Sumner AE, Reynolds JC, Sebring NG, et al. A better index of body adiposity. Obesity. 2011;19(5):1083-9.

8. Mosteller R. Simplified calculation of body surface area. New Engl J Med. 1987;317:1098.
