# Supplementary material for: Predicting the 10-year incidence of dyslipidemia based on novel anthropometric indices, using data mining
Source: Lipids Health Dis. 2024 Jan 31;23:33. doi: 10.1186/s12944-024-02006-2 (PMC10829243; doi:10.1186/s12944-024-02006-2)
Supplement: Supplementary file 2 — Additional file 2: Supplementary Appendix 2. The association between anthropometric measurements and dyslipidemia using Logistic Regression (LR) Model. Table 1.Supp. Best Cut-off values of anthropometric indices maximizing sensitivity plus specificity using receiver operating characteristic analysis. Table 2.Supp. Association of the binary anthropometric indices with incident dyslipidemia based on optimal cutoff point. Figure 1.Supp. Model A based on optimal cutoff point, in Table 4. Figure 2.Supp. Model B based on optimal cutoff point, in Table 4. [file 12944_2024_2006_MOESM2_ESM.docx]

**Supplementary Appendix 2: The association between anthropometric measurements and dyslipidemia using Logistic Regression (LR) Model**

Furthermore, the ROC analysis was conducted to find the optimal cutoff points for each anthropometric variable. The BMI, BRI, and VAI (they are in Model A as well) had the greatest discriminatory power compared to other variables with AUC = 66.3%, 65%, and 66.4% along with their 95% confidence intervals respectively. In each obtained optimal cutoff point, the Youden index and its corresponding sensitivity and specificity for incident dyslipidemia were at their maximum values (Table 1.Supp).

Table 2.Supp shows the association between binary anthropometric variables, with guidelines for the cutoff in Table 3. For instance, the odds of dyslipidemia for participants with VAI in model A, above 1.02 is 2.59 times the odds of non-dyslipidemia significantly (*P*< 0.001) (OR: 2.59, (95% CI: 1.11, 3.20)). Similarly in model B, VAI is a significant predictor of dyslipidemia (OR: 2.11, (95% CI: 1.64, 2.73)). BRI is not significant in either model A or B, although it is highly significant as a single predictor. BMI is a significant predictor in model A (*P* = 0.015) (OR: 1.35, (95% CI: 1.06, 1.73), but it is not significant in model B. Again it was found that model B has a significantly better fit than model A in terms of deviance (2195.5 vs 1504.0, *P*< 0.001).

The AUC value of ROC was obtained at nearly 70% which shows almost high predictability of the logistic regression model. The ROC curve in train and test are shown in Figures 1.Supp and 2.Supp for Models A and B, respectively. Because of the fact BRI, BMI, and AVI are defined by some mathematical formulas, it is needed to pay full attention to the functionality of these variables in the models (Nonlinear). With the help of ROC curves, the cutoff used to make the best accuracy and high AUC. Therefore all of the cutoffs in Table 1.Supp were found based on optimal ROC.

**Table 1.Supp:** Best Cut-off values of anthropometric indices maximizing sensitivity plus specificity using receiver operating characteristic analysis.

| Variable | Best cutoff points | Sensitivity | Specificity | Youden index | LR+ | LR- | AUC (95% CI) |
| --- | --- | --- | --- | --- | --- | --- | --- |
| BMI | 28.04 | 65.5 | 60.1 | 0.255 | 1.64 | 0.57 | 0.663 (0.651, 0.675) |
| BAI | 31.71 | 66.7 | 47.2 | 0.139 | 1.26 | 0.70 | 0.598 (0.586, 0.610) |
| BSA | 1.76 | 63.1 | 51.1 | 0.142 | 1.29 | 0.72 | 0.597 (0.585, 0.609) |
| C-Index | 1.28 | 66.0 | 44.0 | 0.100 | 1.18 | 0.77 | 0.567 (0.555, 0.579) |
| BRI | 5.2 | 65.9 | 57.2 | 0.230 | 1.54 | 0.60 | 0.650 (0.638, 0.661) |
| LAP | 26.64 | 62.78 | 61.42 | 0.2420 | 1.63 | 0.61 | 0.649 (0.626, 0.671) |
| VAI | 1.02 | 70.36 | 54.84 | 0.2520 | 1.56 | 0.54 | 0.664 (0.641, 0.686) |
| AVI | 16.3 | 60.48 | 52.5 | 0.1297 | 1.27 | 0.75 | 0.579 (0.555, 0.602) |
| WWI | 11.18 | 49.45 | 61 | 0.1044 | 1.27 | 0.83 | 0.599 (0.535, 0.582) |
| Abbreviations: BRI (body roundness index), VAI (Visceral Adiposity Index), LAP (Lipid Accumulation Product), AVI (Abdominal volume index), WWI (weight-adjusted-waist index), BMI (body mass index), BAI (Body Adiposity Index), ABSI (Body Shape Index), BAI (Body Adiposity Index), C-Index (conicity index) and BSA (body surface area). | | | | | | | |

**Table 2.Supp:** Association of the binary anthropometric indices with incident dyslipidemia based on optimal cutoff point

| Variable | | Crude  OR(95%CI) | *P*-value | Model A  OR (95% CI) | *P*-value | VIF | Model B  OR (95% CI) | *P*-value | VIF |
| --- | --- | --- | --- | --- | --- | --- | --- | --- | --- |
| VAI | | 2.87 (2.35, 3.52) | <0.001 | 2.59 (1.11, 3.20) | <0.001 | 1.06 | 2.11 (1.64, 2.73) | <0.001 | 1.11 |
| BRI | | 1.74 (1.44, 2.17) | <0.001 | 1.18 (0.92, 1.50) | 0.181 | 1.46 | 1.06 (0.77, 1.45) | 0.690 | 1.69 |
| BMI | | 1.83 (1.50, 2.23) | <0.001 | 1.35 (1.06, 1.73) | 0.015 | 1.44 | 1.17 (0.85, 1.61) | 0.311 | 1.65 |
| Sensitivity | **Train** | - | | 82% | | | 81% | | |
|  | **Test** | - | | 81% | | | 79% | | |
| Deviance = -2LLR | | - | | 2195.53 | | | 1504.02 | | |
| P value of deviance | | - | | <0.001 | | | | | |
| The model A contains binary VAI, BRI and BMI  The model B adjusted based on PAL, Age, Sex, Anxiety Score, Depression Score, Smoking status, percent of daily energy intake from fat, percent of daily energy intake  from carbohydrate and percent of daily energy intake from protein.  Abbreviations: BRI (body roundness index), VAI (Visceral Adiposity Index), BMI (body mass index), and PAL (physical activity level). | | | | | | | | | |

| \|  \| **dislipidemia_out** \| **AUC** \| \| --- \| --- \| --- \| \|  \| **Non dislipidemia** \| 0.6436 \| \|  \| **dislipidemia** \| 0.6436 \|    | \|  \| **dislipidemia** \| **AUC** \| \| --- \| --- \| --- \| \|  \| **Non- dislipidemia** \| 0.6601 \| \|  \| **dislipidemia** \| 0.6601 \|    |
| --- | --- | --- | --- | --- | --- | --- | --- | --- | --- | --- | --- | --- | --- | --- | --- | --- | --- | --- | --- |
| Train | Test |
| **Figure 1.Supp:** Model A based on optimal cutoff point, in table 4 | |

| \|  \| **dislipidemia_out** \| **AUC** \| \| --- \| --- \| --- \| \|  \| **Non dislipidemia** \| 0.68 \| \|  \| **dislipidemia** \| 0.68 \|    | \|  \| **dislipidemia** \| **AUC** \| \| --- \| --- \| --- \| \|  \| **Non- dislipidemia** \| 0.69 \| \|  \| **dislipidemia** \| 0.69 \|    |
| --- | --- | --- | --- | --- | --- | --- | --- | --- | --- | --- | --- | --- | --- | --- | --- | --- | --- | --- | --- |
| Train | Test |
| **Figure 2.Supp:** Model B based on optimal cutoff point, in table 4 | |
